# Risk factors for intraoperative blood loss in resection of intracranial meningioma: Analysis of 530 cases

**DOI:** 10.1371/journal.pone.0291171

**Published:** 2023-09-08

**Authors:** Chenghong Wang, Peng Li

**Affiliations:** 1 Department of Anesthesiology, Sichuan Provincial People’ Hospital, University of Electronic Science and Technology of China, Chengdu, China; 2 Chinese Academy of Sciences Sichuan Translational Medicine Research Hospital, Chengdu, China; Shaanxi Provincial People’s Hospital, CHINA

## Abstract

**Purpose:**

Excision of intracranial meningioma has been associated with major intraoperative blood loss (IBL). The objective of the study was to identify factors affecting IBL during removal of meningioma.

**Methods:**

We retrospectively studied medical records of 530 adult patients who underwent surgery for intracranial meningioma at Sichuan Provincial People’s Hospital between September 2018 and May 2022. We obtained the following data from each patient’s medical chart: age, sex, height, weight, comorbidities, blood pressure, history of smoking and alcohol, imaging examination findings, pathologic diagnosis, albumin, creatinine, calcium, magnesium, hemoglobin (Hb), hematocrit, platelet count, activated partial thromboplastin time, international normalized ratio, fibrinogen concentration and blood transfusion. Univariate and multivariate analyses were performed to identify risk factors for greater IBL during removal of intracranial meningioma.

**Results:**

A total of 530 patients were included in our study. Univariate analysis revealed that sex (*p* = 0.004), two-dimensional (2D) tumor area (*p* < 0.001), sinus involvement (*p* = 0.014), World Health Organization grade (*p* = 0.015), preoperative albumin level (*p* = 0.032), preoperative Hb level (*p* = 0.001) and preoperative platelet count (*p* = 0.004) were significantly associated with greater IBL. Multivariate analysis revealed that greater 2D tumor area (*p* < 0.001), higher preoperative albumin concentration (*p* = 0.029) and higher preoperative platelet count (*p* = 0.03) were independent risk factors for greater IBL in resection of intracranial meningioma.

**Conclusion:**

Larger tumor size, higher preoperative albumin concentration and higher preoperative platelet count were identified as independent risk factors for greater IBL in resection of intracranial meningioma.

## Introduction

Intracranial meningiomas are among the most common primary central nervous system tumors, accounting for more than two-fifths of all primary intracranial tumors [1]. The incidence of intracranial meningiomas is approximately 2.3–5.5 cases per 100 000 individuals, the range inclusive of both incidental and autopsy findings [2]. Patients may be surgically cured once gross total resection is performed because the majority of meningiomas are benign. If subtotal or partial resection is performed due to tight adhesion of tumor to important nervous tissue or blood vessels, a smaller tumor burden may be addressed by other adjuvant treatment, including stereotactic radiotherapy. Thus, surgical resection is one of the primary current standard options for the treatment of intracranial meningiomas. However, excision of intracranial meningioma is frequently associated with excessive blood loss due to significant vascularity of tumor [3]. Moreover, meningioma may induce local tissue plasminogen activator and result in hyperfibrinolysis and deranged coagulogram during surgery [4].

Intraoperative blood loss (IBL) is associated with perioperative complications including hemodynamic instability, anemia, thrombocytopenia, coagulation disorder, hypothermia, infection, systemic inflammation, postoperative intracranial hemorrhage and poor neurological outcome in patients undergoing cranial operation [5, 6]. Need for blood or blood product transfusion is more frequent in patients who sustain massive IBL. Moreover, excessive IBL during cranial surgery is strongly associated with worse prognosis reflected by prolonged duration of mechanical ventilation, intensive care unit stay and hospital stay and even increase in mortality [5, 7]. However, there is a paucity of large sample studies focusing on independent risk factors for massive IBL in patients undergoing surgery for intracranial meningiomas. Therefore, this study was designed to investigate preoperative risk factors associated with greater IBL during resection of intracranial meningiomas to help surgical team identify patients who are at high risk of excessive IBL and require additional medical interventions to minimize blood loss.

## Materials and methods

### Patients and data

We retrospectively reviewed the medical charts of adult patients with intracranial meningioma who underwent cranial surgery at Sichuan Provincial People’s Hospital between September 2018 and May 2022. The inclusion criteria were 1) age ≥ 18 years, 2) single intracranial meningioma detected by preoperative imaging examination and then surgically resected, and 3) meningioma confirmed by pathologic examination. Cases were excluded if 1) they were younger than 18 years old; 2) biopsy rather than surgical removal was performed; 3) the patients had a history of bleeding diathesis or received antithrombotic therapy; and 4) data were incomplete. This study was conducted during November 2022 to April 2023. This study was performed in line with the principles of the Helsinki Declaration. Approval was granted by the Ethics Committee of Sichuan Provincial People’ Hospital. Individual informed consent form for inclusion was waived by the ethics committee because of our retrospective design and because identities of patients were hidden during this study. Authors had no access to information that could identify individual participants during or after data collection.

Based on our inclusion and exclusion criteria, a total of 530 cases were enrolled in our study. We collected the following perioperative information from medical records: age, sex, height, weight, comorbidities, blood pressure, history of smoking and alcohol, findings of imaging examination, pathologic diagnosis, albumin, creatinine (Cr), calcium, magnesium, hemoglobin (Hb), hematocrit (HCT), platelet count, activated partial thromboplastin time (APTT), international normalized ratio (INR), fibrinogen concentration, blood transfusion and outcome. Preoperative head magnetic resonance imaging (MRI) was routinely performed unless there was contraindication and MRI was replaced by CT. The two-dimensional (2D) tumor area, which was used to quantify the tumor size, was calculated as the product of the largest tumor diameter and its largest perpendicular diameter on the same slide [8]. IBL was calculated using Modified Gross formula if there was no intraoperative blood transfusion [9]. Blood loss = Estimated blood volume (EBV) × [Initial hematocrit (iHCT)—Final hematocri (fHCT)]/Mean hematocrit (mHCT). EBV = Body weight × [70 (for females) or 75 (for males)] (ml/kg). Formula by Brecher et al. was utilized if the patient received intraoperative blood transfusion [10]. Blood loss = Blood loss_1_ + Blood loss_2_. Blood loss_1_ = EBV × ln [iHCT/minimal hematocrit (min HCT)]. Blood Loss_2_ = [Red blood cell_1_ (RBC_1_)—RBC_2_]/min HCT. RBC_1_ represents total amount of red blood cell transfused. RBC_2_ = (fHCT—min HCT) × EBV. The median IBL was used as the threshold for a high *vs* low IBL value. Surgical procedures

Neuronavigation might be used to help surgeons confirm the location of incision and surgical bone window before removal of non-skull base meningiomas. A lumbar drain might be placed before excision of giant skull base meningiomas. Electrophysiological monitoring was performed if necessary. After surgical bone window was made and dura mater was opened, cerebrospinal fluid was released by opening cisternal arachnoid membranes to promote brain relaxation. Most of the meningiomas were resected under microscope or endoscope. If possible, tumor base was separated from attachment site before hypervascular tumor was removal. Gross total resection of meningioma with coagulation or excision of its dural attachment was considered unless it was unable to be achieved due to tight adhesion of the lesion to important nervous tissue or blood vessels. Electrocoagulation was usually utilized to manage arterial hemorrhage while hemostasis by compression was used to control venous bleeding. Blood salvage was applied in patients with a tendency to sustain massive bleeding. Absorbable hemostat, gelatin sponge or fibrin sealant was used if necessary. Before the dura mater was closed, neurosurgeons made sure no visible bleeding site existed in surgical field.

### General anesthesia

On admission to the operating room, patients were placed in the supine position and routinely monitored by cardiac electrical activity, noninvasive blood pressure measurement, and pulse oximetry. In most cases, arterial catheter and central venous catheter were placed. Sedation monitoring and temperature monitoring were performed intraoperatively if necessary. General anesthesia was induced with sufentanil, propofol and cisatracurium. After intubation, mechanical ventilation was adjusted to have the end-tidal carbon dioxide between 30 and 40 mmHg. Anesthesia was maintained with sevoflurane, propofol or dexmedetomidine, remifentanil, and intermittent doses of cisatracurium. Arterial blood gas analysis was performed as required. Intraoperative data including vital signs, pulse oxygen saturation, findings of blood gas analysis and information about blood transfusion were recorded.

### Statistical analysis

The statistical analyses were performed with SPSS statistical software version 26. Continuous variables were presented as numbers with percentages, whereas continuous data were presented as median with interquartile range (IQR) because all continuous data were distributed nonnormally in our study, which was confirmed by Kolmogorov-Smirnov test. Categorical variables were compared using chi-square test while Wilcoxon-Mann-Whitney test was applied to compare continuous data. Several variables that were identified as being significant in the univariate analysis were included in the multivariate analysis, which was performed using a logistic regression. Two-sided *p* values <0.05 were considered to indicate statistical significance.

## Results

### Baseline characteristics and IBL

The medical records of 542 patients were reviewed. As shown in Table 1, a total of 530 patients, including 137 men and 393 women, were enrolled in our study. Two, 1 and 9 patients were excluded because of age < 18 years old, procedure of biopsy and incompleteness of data, respectively. The median patient age was 55 years (IQR, 48–64 years). The median 2D tumor area was 12.05 cm^2^ (IQR, 6.25–20.83 cm^2^). More than half (52.5%) of the meningiomas were located at skull base. The histology of 92.26% intracranial meningiomas was World Health Organization (WHO) Grade I, 7.36% WHO Grade II and 0.38% WHO Grade III. The median IBL [691.43 ml (IQR, 340.65–1106.34 ml)] was used as the threshold for a high *vs* low IBL value.

**Table 1:** Patient characteristics.

Variables	*n* (%) or median (IQR)
Age, year	55 (48–64)
Sex	
Male	137 (25.8%)
Female	393 (74.2%)
BMI	23.63 (21.51–25.78)
Diabetes	39 (7.4%)
Hypercholesterolemia	129 (24.3%)
Hypertension	118 (22.3%)
SBP, mmHg	122 (113–130)
DBP, mmHg	74 (67–79)
APTT, s	26.8 (25.4–28.3)
INR	0.975 (0.93–1.01)
Fibrinogen concentration, g/L	2.77 (2.41–3.2)
Platelet count, × 10^9^ /L	186 (147–231)
2D tumor area, cm^2^	12.05 (6.25–20.83)
WHO grade	
I	489 (92.26%)
II	39 (7.36%)
III	2 (0.38%)

Abbreviations: APTT, activated partial thromboplastin time; BMI, body mass index; DBP, diastolic blood pressure; INR, international normalized ratio; IQR, interquartile range; SBP, systolic blood pressure; WHO, World Health Organization.

### Risk factors for high IBL during resection of intracranial meningioma

As shown in Tables 2 and 3, the differences between cases with higher IBL and those with lower IBL were compared. Univariate analysis revealed that higher IBL was significantly associated with sex (male; odds ratio [OR], 1.782; 95% confidence interval [CI], 1.199–2.647; *p* = 0.004, chi-square test), lager 2D tumor area (median, 16.45; IQR, 9.57–28.71 vs. median, 9; IQR, 4.83–15.99 cm^2^, respectively; *p* < 0.001, Wilcoxon-Mann-Whitney test), sinus involvement (OR, 1.545; 95% CI, 1.092–2.185; *p* = 0.014, chi-square test), World Health Organization grade II or III (OR, 2.290; 95% CI, 1.159–4.526; *p* = 0.015, chi-square test), preoperative albumin level (median, 40; IQR, 38.15–42.1 vs. median, 39.3; IQR, 37.6–41.45 g/L, respectively; *p* = 0.032, Wilcoxon-Mann-Whitney test), preoperative Hb level (median, 133; IQR, 124–144 vs. median, 129; IQR, 120–138 g/L, respectively; *p* = 0.001; Wilcoxon-Mann-Whitney test) and preoperative platelet count (median, 191; IQR, 157.5–237.5 vs. median, 181; IQR, 141.5–224 ×10^9^/L, respectively; *p* = 0.004; Wilcoxon-Mann-Whitney test). Multivariate analysis revealed that greater 2D tumor area (*p* < 0.001; β = 1.081; 95% CI, 1.059–1.103), higher preoperative albumin concentration (*p* = 0.029; β = 1.065; 95% CI, 1.007–1.127) and higher preoperative platelet count (*p* = 0.03; β = 1.003; 95% CI, 1.000–1.007) were independent risk factors for greater IBL in adult patients undergoing resection of intracranial meningioma (Table 3).

**Table 2:** Univariate analysis of association between each factor and IBL.

Variables	IBL, n (%) or median (range)		*p* value
	High (n = 265)	Low (n = 265)	
Age	55 (48–64)	54 (49–63)	0.573
Sex			0.004*
Male	83 (31.3%)	54 (20.4%)	
Female	182 (68.7%)	211 (79.6%)	
BMI	23.81 (21.48–25.79)	23.56 (21.64–25.78)	0.685
History of smoke			0.215
Yes	43 (16.2%)	33 (12.5%)	
No	222 (83.8%)	232 (87.5%)	
History of alcohol			0.074
Yes	26 (9.8%)	15 (5.7%)	
No	239 (90.2%)	250 (94.3%)	
Diabetes			0.868
Present	20 (7.5%)	19 (7.2%)	
Absent	245 (92.5%)	246 (92.8%)	
Hypercholesterolemia			0.266
Present	70 (26.4%)	59 (22.3%)	
Absent	195 (73.6%)	206 (77.7%)	
Hypertension			0.676
Present	57 (21.5%)	61 (23%)	
Absent	208 (78.5%)	204 (77%)	
SBP, mmHg	122 (113–130)	122 (113–130)	0.869
DBP, mmHg	74 (67–79)	73 (67–79)	0.663
2D tumor area, cm^2^	16.45 (9.57–28.71)	9 (4.83–15.99)	<0.001*
Tumor location			0.602
Skull base	142 (53.6%)	136 (51.3%)	
Non-skull base	123 (46.4%)	129 (48.7%)	
Tumor location			0.661
Midline	111 (41.9%)	116 (43.8%)	
Lateral	154 (58.1%)	149 (56.2%)	
Attachment to sinus			0.014*
Yes	126 (47.5%)	98 (37%)	
No	139 (52.5%)	167 (63%)	
WHO grade			0.015*
I	237 (89.4%)	252 (95.1%)	
II or III	28 (10.6%)	13 (4.9%)	
Preoperative albumin concentration, g/L	40 (38.15–42.1)	39.3 (37.6–41.45)	0.032*
Preoperative Cr level, umol/L	57.8 (50–66.3)	55.3 (49.15–63.95)	0.058
Preoperative calcium level, mmol/L	2.23 (2.16–2.31)	2.22 (2.16–2.29)	0.122
Preoperative magnesium level, mmol/L	0.9 (0.85–0.95)	0.89 (0.85–0.94)	0.215
Preoperative Hb level	133 (124–144)	129 (120–138)	0.001*
Preoperative APTT, s	26.9 (25.4–28.65)	26.6 (25.35–27.9)	0.244
Preoperative INR	0.98 (0.93–1.02)	0.97 (0.93–1.01)	0.708
Preoperative fibrinogen concentration, g/L	2.8 (2.45–3.26)	2.71 (2.38–3.16)	0.059
Preoperative platelet count, × 10^9^ /L	191 (157.5–237.5)	181 (141.5–224)	0.004*

*p <0.05

Abbreviations: APTT, activated partial thromboplastin time; BMI, body mass index; Cr, creatinine; DBP, diastolic blood pressure; Hb, hemoglobin; INR, international normalized ratio; IQR, interquartile range; POH, postoperative hemorrhage; SBP, systolic blood pressure; WHO, World Health Organization.

**Table 3:** Multivariate analysis of factors associated with IBL.

Variables	OR (95% CI)	*p* value
Sex	1.089 (0.652–1.819)	0.744
2D tumor area	1.081 (1.059–1.103)	<0.001*
Attachment to sinus	1.334 (0.906–1.966)	0.145
WHO grade	1.431 (0.669–3.061)	0.355
Preoperative albumin concentration	1.065 (1.007–1.127)	0.29*
Preoperative Hb level	1.01 (0.996–1.023)	0.149
Preoperative platelet count	1.003 (1.000–1.007)	0.03*

*p <0.05

Abbreviations: Hb, hemoglobin; OR, odds ratio.

## Discussion

Patients undergoing resection of intracranial meningioma frequently present high risks of massive IBL, which is associated with increased morbidity and mortality [5, 7]. Thus, reducing IBL during excision of intracranial meningioma has been one of the main concerns of surgical team. There are potentially effective approaches to reduce IBL, including ethmoidal artery ligation [11], extradural devascularization [12], temporary external carotid artery clamping [13], embolization for meningioma [14], intratumoral hydrogen peroxide injection [15], radio frequency thermocoagulation [16] and hemostatic agents (e.g., tranexamic acid) [17, 18]. However, there is a lack of consensus regarding the ideal intracranial meningiomas for these additional medical interventions. Hence, preoperative predictors for significant IBL may shed light on this topic. In the present study, larger tumor size, higher preoperative albumin concentration and higher preoperative platelet count were identified as significant independent risk factors for greater IBL in adult patients undergoing resection of intracranial meningioma.

The reported mean or median amount of IBL during surgery for intracranial meningioma varied from about 300 ml to 1100 ml in the literature [4, 17–20]. The median IBL was 691.43 ml in our study. IBL varied a lot in the literature possibly due to different samples and differences in tumor size, surgical techniques, perioperative management and methods used to estimate the amount of IBL among studies.

Our findings revealed that patients with large intracranial meningioma were more likely to sustain significant IBL. Similarly, Tabibkhooei et al. reported that the size of intracranial meningioma was positively correlated with the volume of IBL [20]. Moreover, large intracranial meningioma was a risk factor for intraoperative red blood cell transfusion [21]. The association between tumor size and IBL might be explained as follows. First, during resection of large tumor, large surgical field might lead to greater volume of IBL. Second, giant meningioma was often high vascularized and more likely to enhance large vessels. Third, surgical time was longer in excision of large tumor and IBL increased with the prolongation of surgical time. Moreover, brain edema caused by giant meningioma and brain injury caused by excessive retraction in resection of giant lesion might increase IBL. Therefore, further studies are necessary to investigate how to prevent massive IBL during removal of giant meningioma. Moreover, these findings emphasize the importance of early treatment of intracranial meningioma.

Higher preoperative albumin concentration was identified as an independent risk factor for IBL greater than 691.43 ml in patients undergoing resection of intracranial meningioma in the present study. It was reported in the literature that albumin induced an impairment of platelet aggregation [22, 23]. Gresele et al. demonstrated that serum albumin might promote the formation of prostaglandin D2 and induce thromboxane synthase inhibition, which resulted in the impairment of platelet aggregation [22]. However, albumin was found to increase platelet adhesion to collagen and fibrinogen in patients with type 2 diabetes [24]. Paar et al. reported that serum albumin might impair thrombin generation and clot formation under experimental conditions [25]. Kim et al. revealed that there were inverse correlations between albumin concentration and von Willebrand factor level in continuous ambulatory peritoneal dialysis patients [26]. Therefore, the effect of serum albumin on platelet and coagulation seemed various and complicated, which should be considered when fluid therapy was required during removal of intracranial meningioma. Li et al. investigated the effects of different colloid infusions on platelet aggregation and coagulation during elective brain tumor surgery [27]. The findings revealed that 5% albumin infusion decreased platelet aggregation while hydroxyethyl starch affected coagulation at lower volumes [27]. Sigurjonsson demonstrated that the effect of 5% albumin infusion on APTT, INR and clot strength was less compared with 6% dextran-70 [28]. Therefore, colloid infusions during removal of intracranial meningioma should be individualized.

It was well known that functioning platelets were essential for surgical hemostasis. However, our findings revealed that preoperative platelet count was higher in patients who sustained large volume IBL during removal of intracranial meningioma. The effect of platelet count on hemostasis might be limited in our sample because the platelet counts in both group were in normal range. We hypothesized that the association between platelet and IBL might be partially attributed to a potential pathogenic feedback loop between platelet and tumor [29–31]. For example, Interleukin-6, which could be produced by meningioma [32], might increase the production of platelet and facilitate tumor angiogenesis [29, 33], which might be associated with significant IBL. Moreover, platelet might promote tumor angiogenesis by releasing angiogenic proteins, lipids, growth factors (e.g., vascular endothelial growth factor), cytokines and proteases [34]. Thus, further research were required to investigate the interaction between platelet and the biological behavior of meningioma.

Our study has some limitations. First, this is a single-center, retrospective study and admission bias may present in our sample. Second, due to the incompleteness of data in some patients, we used 2D tumor area rather than tumor volume to describe tumor size and some imaging factors which might be associated with IBL in resection of intracranial meningioma were not included in our study [35, 36]. Furthermore, a margin of error might exist in this study because IBL was calculated using formulas. Therefore, our findings need to be confirmed by other multicenter prospective studies.

## Conclusion

Large tumor size, higher preoperative albumin concentration and higher preoperative platelet count were identified as independent risk factors for significant IBL in adult patients undergoing resection of intracranial meningioma. These findings need to be confirmed by multicenter prospective studies. Nevertheless, a rigorous surgical technique remains one of the most important way to minimize IBL during excision of intracranial meningioma.

## Supporting information

S1 Checklist STROBE statement—checklist of items that should be included in reports of observational studies. (DOCX)

S1 Dataset (DOCX)
