# Supplementary material for: Risk factors for intraoperative blood loss in resection of intracranial meningioma: Analysis of 530 cases
Source: PLoS One. 2023 Sep 8;18(9):e0291171. doi: 10.1371/journal.pone.0291171 (PMC10490957; doi:10.1371/journal.pone.0291171)
Supplement: S1 Checklist — (DOCX) [file pone.0291171.s001.docx]

STROBE Statement—checklist of items that should be included in reports of observational studies

|  | Item No. | Recommendation | Page  No. | Relevant text from manuscript |
| --- | --- | --- | --- | --- |
| **Title and abstract** | 1 | (*a*) Indicate the study’s design with a commonly used term in the title or the abstract | 2 | We retrospectively studied medical records of 530 adult patients |
|  |  | (*b*) Provide in the abstract an informative and balanced summary of what was done and what was found | 2 | Univariate and multivariate analyses were performed to identify risk factors for greater IBL during removal of intracranial meningioma.  Larger tumor size, higher preoperative albumin concentration and higher preoperative platelet count were identified as independent risk factors for greater IBL in resection of intracranial meningioma. |
| Introduction | | | |  |
| Background/rationale | 2 | Explain the scientific background and rationale for the investigation being reported | 3 | Intraoperative blood loss (IBL) is associated with perioperative complications including hemodynamic instability, anemia, thrombocytopenia, coagulation disorder, hypothermia, infection, systemic inflammation, postoperative intracranial hemorrhage and poor neurological outcome in patients undergoing cranial operation.  Moreover, excessive IBL during cranial surgery is strongly associated with worse prognosis reflected by prolonged duration of mechanical ventilation, intensive care unit stay and hospital stay and even increase in mortality. However, there is a paucity of large sample studies focusing on independent risk factors for massive IBL in patients undergoing surgery for intracranial meningiomas. |
| Objectives | 3 | State specific objectives, including any prespecified hypotheses | 3 | Therefore, this study was designed to investigate preoperative risk factors associated with greater IBL during resection of intracranial meningiomas to help surgical team identify patients who are at high risk of excessive IBL and require additional medical interventions to minimize blood loss. |
| Methods | | | |  |
| Study design | 4 | Present key elements of study design early in the paper | 4, 5 and 6 | Based on our inclusion and exclusion criteria, a total of 530 cases were enrolled in our study. We collected the following perioperative information from medical records: age, sex, height, weight, comorbidities, blood pressure, history of smoking and alcohol, findings of imaging examination, pathologic diagnosis, albumin, creatinine (Cr), calcium, magnesium, hemoglobin (Hb), hematocrit (HCT), platelet count, activated partial thromboplastin time (APTT), international normalized ratio (INR), fibrinogen concentration, blood transfusion and outcome.  The median IBL was used as the threshold for a high vs low IBL value.  Several variables that were identified as being significant in the univariate analysis were included in the multivariate analysis, which was performed using a logistic regression. |
| Setting | 5 | Describe the setting, locations, and relevant dates, including periods of recruitment, exposure, follow-up, and data collection | 4 | We retrospectively reviewed the medical charts of adult patients with intracranial meningioma who underwent cranial surgery at Sichuan Provincial People’s Hospital between September 2018 and May 2022.  Based on our inclusion and exclusion criteria, a total of 530 cases were enrolled in our study. We collected the following perioperative information from medical records: age, sex, height, weight, comorbidities, blood pressure, history of smoking and alcohol, findings of imaging examination, pathologic diagnosis, albumin, creatinine (Cr), calcium, magnesium, hemoglobin (Hb), hematocrit (HCT), platelet count, activated partial thromboplastin time (APTT), international normalized ratio (INR), fibrinogen concentration, blood transfusion and outcome. |
| Participants | 6 | (*a*) *Cohort study*—Give the eligibility criteria, and the sources and methods of selection of participants. Describe methods of follow-up  *Case-control study*—Give the eligibility criteria, and the sources and methods of case ascertainment and control selection. Give the rationale for the choice of cases and controls  *Cross-sectional study*—Give the eligibility criteria, and the sources and methods of selection of participants | 4 | We retrospectively reviewed the medical charts of adult patients with intracranial meningioma who underwent cranial surgery at Sichuan Provincial People’s Hospital between September 2018 and May 2022. The inclusion criteria were 1) age ≥ 18 years, 2) single intracranial meningioma detected by preoperative imaging examination and then surgically resected, and 3) meningioma confirmed by pathologic examination. Cases were excluded if 1) they were younger than 18 years old; 2) biopsy rather than surgical removal was performed; 3) the patients had a history of bleeding diathesis or received antithrombotic therapy; and 4) data were incomplete. We collected the following perioperative information from medical records: age, sex, height, weight, comorbidities, blood pressure, history of smoking and alcohol, findings of imaging examination, pathologic diagnosis, albumin, creatinine (Cr), calcium, magnesium, hemoglobin (Hb), hematocrit (HCT), platelet count, activated partial thromboplastin time (APTT), international normalized ratio (INR), fibrinogen concentration, blood transfusion and outcome.  IBL was calculated using Modified Gross formula if there was no intraoperative blood transfusion. |
|  |  | (*b*) *Cohort study*—For matched studies, give matching criteria and number of exposed and unexposed  *Case-control study*—For matched studies, give matching criteria and the number of controls per case |  |  |
| Variables | 7 | Clearly define all outcomes, exposures, predictors, potential confounders, and effect modifiers. Give diagnostic criteria, if applicable | 5 and 6 | The median IBL was used as the threshold for a high vs low IBL value.  Most of the meningiomas were resected under microscope or endoscope. If possible, tumor base was separated from attachment site before hypervascular tumor was removal. Gross total resection of meningioma with coagulation or excision of its dural attachment was considered unless it was unable to be achieved due to tight adhesion of the lesion to important nervous tissue or blood vessels. Electrocoagulation was usually utilized to manage arterial hemorrhage while hemostasis by compression was used to control venous bleeding. Blood salvage was applied in patients with a tendency to sustain massive bleeding.  Several variables that were identified as being significant in the univariate analysis were included in the multivariate analysis, which was performed using a logistic regression. |
| Data sources/ measurement | 8* | For each variable of interest, give sources of data and details of methods of assessment (measurement). Describe comparability of assessment methods if there is more than one group | 4 and 5 | IBL was calculated using Modified Gross formula if there was no intraoperative blood transfusion [9]. Blood loss = Estimated blood volume (EBV) × [Initial hematocrit (iHCT) - Final hematocri (fHCT)]/Mean hematocrit (mHCT). EBV = Body weight × [70 (for females) or 75 (for males)] (ml/kg). Formula by Brecher et al. was utilized if the patient received intraoperative blood transfusion [10]. Blood loss = Blood loss1 + Blood loss2. Blood loss1 = EBV × ln [iHCT/minimal hematocrit (min HCT)]. Blood Loss2 = [Red blood cell1 (RBC1) - RBC2]/min HCT. RBC1 represents total amount of red blood cell transfused. RBC2 = (fHCT - min HCT) × EBV. The median IBL was used as the threshold for a high vs low IBL value. |
| Bias | 9 | Describe any efforts to address potential sources of bias | 4 and 6 | We collected the following perioperative information from medical records: age, sex, height, weight, comorbidities, blood pressure, history of smoking and alcohol, findings of imaging examination, pathologic diagnosis, albumin, creatinine (Cr), calcium, magnesium, hemoglobin (Hb), hematocrit (HCT), platelet count, activated partial thromboplastin time (APTT), international normalized ratio (INR), fibrinogen concentration, blood transfusion and outcome. Continuous variables were presented as numbers with percentages, whereas continuous data were presented as median with interquartile range (IQR) because all continuous data were distributed nonnormally in our study, which was confirmed by Kolmogorov-Smirnov test. Categorical variables were compared using chi-square test while Wilcoxon-Mann-Whitney test was applied to compare continuous data. Several variables that were identified as being significant in the univariate analysis were included in the multivariate analysis, which was performed using a logistic regression. |
| Study size | 10 | Explain how the study size was arrived at | 4 | We retrospectively reviewed the medical charts of adult patients with intracranial meningioma who underwent cranial surgery at Sichuan Provincial People’s Hospital between September 2018 and May 2022. |

Continued on next page

| Quantitative variables | 11 | Explain how quantitative variables were handled in the analyses. If applicable, describe which groupings were chosen and why | 6 | Continuous variables were presented as numbers with percentages, whereas continuous data were presented as median with interquartile range (IQR) because all continuous data were distributed nonnormally in our study, which was confirmed by Kolmogorov-Smirnov test. |
| --- | --- | --- | --- | --- |
| Statistical methods | 12 | (*a*) Describe all statistical methods, including those used to control for confounding | 6 | The statistical analyses were performed with SPSS statistical software version 26. Continuous variables were presented as numbers with percentages, whereas continuous data were presented as median with interquartile range (IQR) because all continuous data were distributed nonnormally in our study, which was confirmed by Kolmogorov-Smirnov test. Categorical variables were compared using chi-square test while Wilcoxon-Mann-Whitney test was applied to compare continuous data. Several variables that were identified as being significant in the univariate analysis were included in the multivariate analysis, which was performed using a logistic regression. Two-sided p values <0.05 were considered to indicate statistical significance. |
|  |  | (*b*) Describe any methods used to examine subgroups and interactions |  | N/A |
|  |  | (*c*) Explain how missing data were addressed | 4 | Cases were excluded if 1) they were younger than 18 years old; 2) biopsy rather than surgical removal was performed; 3) the patients had a history of bleeding diathesis or received antithrombotic therapy; and 4) data were incomplete. |
|  |  | (*d*) *Cohort study*—If applicable, explain how loss to follow-up was addressed  *Case-control study*—If applicable, explain how matching of cases and controls was addressed  *Cross-sectional study*—If applicable, describe analytical methods taking account of sampling strategy |  | N/A |
|  |  | (*e*) Describe any sensitivity analyses |  | N/A |
| Results | | | | |
| Participants | 13* | (a) Report numbers of individuals at each stage of study—eg numbers potentially eligible, examined for eligibility, confirmed eligible, included in the study, completing follow-up, and analysed | 6 | The medical records of 542 patients were reviewed. As shown in Table 1, a total of 530 patients, including 137 men and 393 women, were enrolled in our study. |
|  |  | (b) Give reasons for non-participation at each stage | 6 | Two, 1 and 9 patients were excluded because of age < 18 years old, procedure of biopsy and incompleteness of data, respectively. |
|  |  | (c) Consider use of a flow diagram |  | N/A |
| Descriptive data | 14* | (a) Give characteristics of study participants (eg demographic, clinical, social) and information on exposures and potential confounders | 6 | The medical records of 542 patients were reviewed. As shown in Table 1, a total of 530 patients, including 137 men and 393 women, were enrolled in our study. Two, 1 and 9 patients were excluded because of age < 18 years old, procedure of biopsy and incompleteness of data, respectively. The median patient age was 55 years (IQR, 48-64 years). The median 2D tumor area was 12.05 cm2 (IQR, 6.25-20.83 cm2). More than half (52.5%) of the meningiomas were located at skull base. The histology of 92.26% intracranial meningiomas was World Health Organization (WHO) Grade I, 7.36% WHO Grade II and 0.38% WHO Grade III. The median IBL [691.43 ml (IQR, 340.65-1106.34 ml)] was used as the threshold for a high vs low IBL value. |
|  |  | (b) Indicate number of participants with missing data for each variable of interest | 6 | Two, 1 and 9 patients were excluded because of age < 18 years old, procedure of biopsy and incompleteness of data, respectively. |
|  |  | (c) *Cohort study*—Summarise follow-up time (eg, average and total amount) |  | N/A |
| Outcome data | 15* | *Cohort study*—Report numbers of outcome events or summary measures over time | 6 and 8 | The median IBL [691.43 ml (IQR, 340.65-1106.34 ml)] was used as the threshold for a high vs low IBL value.  High (n = 265)  Low (n = 265) |
|  |  | *Case-control study—*Report numbers in each exposure category, or summary measures of exposure |  |  |
|  |  | *Cross-sectional study—*Report numbers of outcome events or summary measures |  |  |
| Main results | 16 | (*a*) Give unadjusted estimates and, if applicable, confounder-adjusted estimates and their precision (eg, 95% confidence interval). Make clear which confounders were adjusted for and why they were included | 7 and 8 | As shown in Tables 2 and 3, the differences between cases with higher IBL and those with lower IBL were compared. Univariate analysis revealed that higher IBL was significantly associated with sex (male; odds ratio [OR], 1.782; 95% confidence interval [CI], 1.199–2.647; *p* = 0.004, chi-square test), lager 2D tumor area (median, 16.45; IQR, 9.57-28.71 vs. median, 9; IQR, 4.83-15.99 cm^2^, respectively; *p* < 0.001, Wilcoxon-Mann-Whitney test), sinus involvement (OR, 1.545; 95% CI, 1.092–2.185; *p* = 0.014, chi-square test), World Health Organization grade II or III (OR, 2.290; 95% CI, 1.159–4.526; *p* = 0.015, chi-square test), preoperative albumin level (median, 40; IQR, 38.15-42.1 vs. median, 39.3; IQR, 37.6-41.45 g/L, respectively; *p* = 0.032, Wilcoxon-Mann-Whitney test), preoperative Hb level (median, 133; IQR, 124-144 vs. median, 129; IQR, 120-138 g/L, respectively; *p* = 0.001; Wilcoxon-Mann-Whitney test) and preoperative platelet count (median, 191; IQR, 157.5-237.5 vs. median, 181; IQR, 141.5-224 ×10^9^/L, respectively; *p* = 0.004; Wilcoxon-Mann-Whitney test). Multivariate analysis revealed that greater 2D tumor area (*p* < 0.001; β = 1.081; 95% CI, 1.059-1.103), higher preoperative albumin concentration (*p* = 0.029; β = 1.065; 95% CI, 1.007-1.127) and higher preoperative platelet count (*p* = 0.03; β = 1.003; 95% CI, 1.000-1.007) were independent risk factors for greater IBL in adult patients undergoing resection of intracranial meningioma (Table 3). |
|  |  | (*b*) Report category boundaries when continuous variables were categorized | 6 | The median IBL [691.43 ml (IQR, 340.65-1106.34 ml)] was used as the threshold for a high *vs* low IBL value. |
|  |  | (*c*) If relevant, consider translating estimates of relative risk into absolute risk for a meaningful time period |  | N/A |

Continued on next page

| Other analyses | 17 | Report other analyses done—eg analyses of subgroups and interactions, and sensitivity analyses |  | N/A |
| --- | --- | --- | --- | --- |
| Discussion | | | | |
| Key results | 18 | Summarise key results with reference to study objectives | 11 | In the present study, larger tumor size, higher preoperative albumin concentration and higher preoperative platelet count were identified as significant independent risk factors for greater IBL in adult patients undergoing resection of intracranial meningioma. |
| Limitations | 19 | Discuss limitations of the study, taking into account sources of potential bias or imprecision. Discuss both direction and magnitude of any potential bias | 13 | Our study has some limitations. First, this is a single-center, retrospective study and admission bias may present in our sample. Second, due to the incompleteness of data in some patients, we used 2D tumor area rather than tumor volume to describe tumor size and some imaging factors which might be associated with IBL in resection of intracranial meningioma were not included in our study [35,36]. Furthermore, a margin of error might exist in this study because IBL was calculated using formulas. Therefore, our findings need to be confirmed by other multicenter prospective studies. |
| Interpretation | 20 | Give a cautious overall interpretation of results considering objectives, limitations, multiplicity of analyses, results from similar studies, and other relevant evidence | 13 | Large tumor size, higher preoperative albumin concentration and higher preoperative platelet count were identified as independent risk factors for significant IBL in adult patients undergoing resection of intracranial meningioma. These findings need to be confirmed by multicenter prospective studies. Nevertheless, a rigorous surgical technique remains one of the most important way to minimize IBL during excision of intracranial meningioma. |
| Generalisability | 21 | Discuss the generalisability (external validity) of the study results | 13 | First, this is a single-center, retrospective study and admission bias may present in our sample. These findings need to be confirmed by multicenter prospective studies. |
| Other information | |  | | |
| Funding | 22 | Give the source of funding and the role of the funders for the present study and, if applicable, for the original study on which the present article is based | 1 | The authors received no specific funding for this work. |

*Give information separately for cases and controls in case-control studies and, if applicable, for exposed and unexposed groups in cohort and cross-sectional studies.

**Note:** An Explanation and Elaboration article discusses each checklist item and gives methodological background and published examples of transparent reporting. The STROBE checklist is best used in conjunction with this article (freely available on the Web sites of PLoS Medicine at http://www.plosmedicine.org/, Annals of Internal Medicine at http://www.annals.org/, and Epidemiology at http://www.epidem.com/). Information on the STROBE Initiative is available at www.strobe-statement.org.
