# Supplementary material for: Risk factors for intraoperative blood loss in resection of intracranial meningioma: Analysis of 530 cases
Source: PLoS One. 2023 Sep 8;18(9):e0291171. doi: 10.1371/journal.pone.0291171 (PMC10490957; doi:10.1371/journal.pone.0291171)
Supplement: S1 Dataset — (DOCX) [file pone.0291171.s002.docx]

DOI: 10.6084/m9.figshare.24039306
